# Investigation of the Thermodynamic and Kinetic Behavior of Acid Dyes in Relation to Wool Fiber Morphology

**DOI:** 10.1021/acsomega.4c00560

**Published:** 2024-06-04

**Authors:** Subhadeep Paul, Andrew Hewitt, Sohel Rana, Parikshit Goswami

**Affiliations:** † Technical Textiles Research Centre, School of Arts and Humanities, University of Huddersfield, Queensgate Huddersfield HD1 3DH, U.K.; ‡ Department of Textile & Fibre Engineering, Indian Institute of Technology Delhi, Hauz Khas New Delhi 110016, India

## Abstract

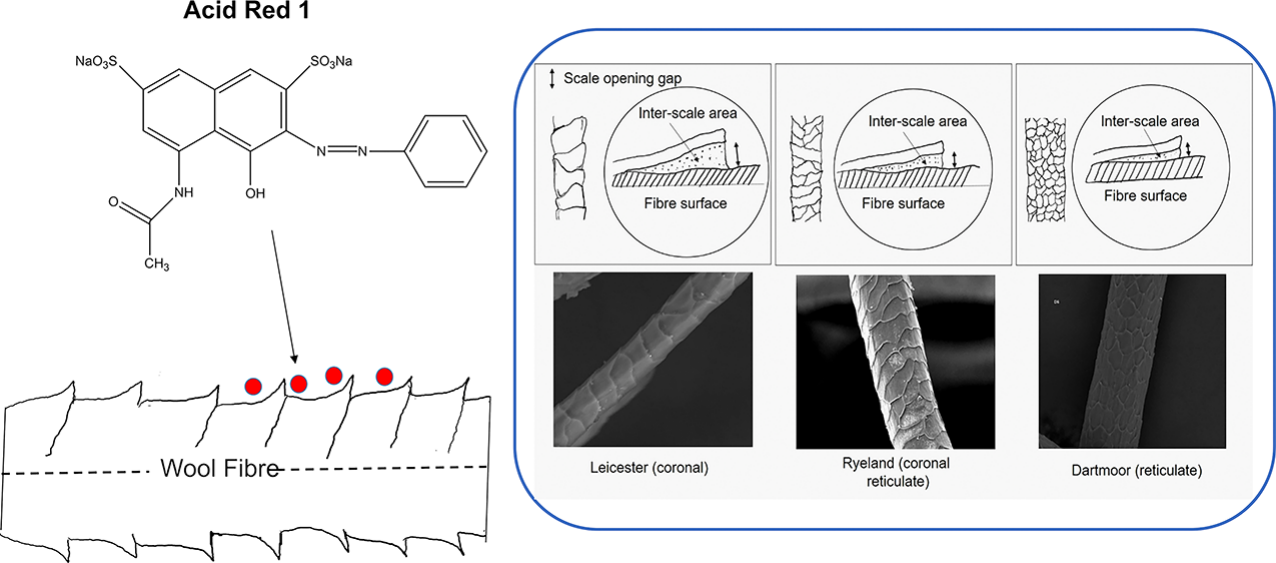

Wool fibers from several different sheep breeds in the UK have very limited applications. The main aim of this study was to establish an understanding of the dye sorption properties of different wool fibers through thermodynamics and kinetics of dyeing using Acid Red 1 dye. Wool fibers from Leicester, Ryeland, and Dartmoor sheep breeds were pretreated (to remove impurities) and dyed using Acid Red 1. Leicester showed 7% higher dye exhaustion than Dartmoor wool fibers (20% on mass of fiber). Dyeing equilibrium results for both Leicester and Dartmoor wool fibers were fitted to Langmuir and Freundlich isotherms, and the theoretical maximum sorption capacities were 164 and 144 mg g^–1^, respectively. Leicester, Ryeland, and Dartmoor also followed the pseudo-second-order reaction kinetics. Thermodynamic parameters like Gibb’s free energy (Δ*G*°) and standard affinity (Δμ°) of the fibers were calculated to understand the interaction of the Acid Red 1 with wool fibers. The difference in dye uptake was explained through the possible involvement of the scale opening gap (surface morphology) of the wool fibers.

## 1 Introduction

Wool fiber obtained from different breeds of sheep has differences in fiber properties such as color of wool, texture, fiber length, crimp, diameter, chemical composition, and macroscopic and microscopic properties.^1−3^ The differences in the macroscopic and microscopic structure of the wool fibers are reflected in the functional behavior of the fibers.^4,5^ Wool is a highly reactive fiber due to the presence of positively and negatively charged functional groups in the form of acidic (−COOH) and basic (−NH_2_) groups.^6^ Wool is a highly amorphous fiber (among all natural fibers) and the presence of different amino acids contributes to its high sorption properties (water, dyes, volatile organic compounds, and other chemicals) as do the hydroxyl and thiol groups present in wool fiber.^7−11^ Investigation of sorption behavior of acid dyes on the surface and in the core of the wool fiber reflects the chemical reactivity of the wool.^12^

**Figure 1:**
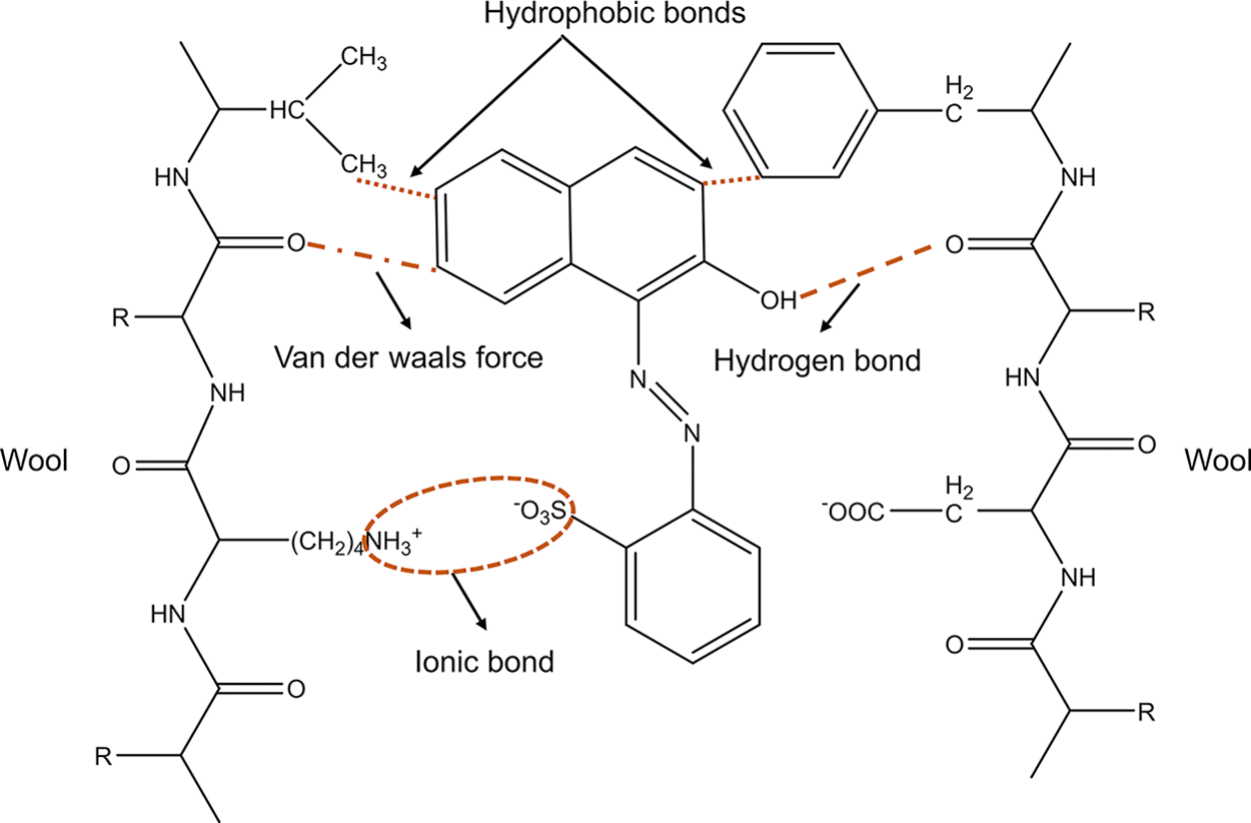
Different types of interaction between acid dye molecule and wool fiber.

Acid (nonmetallized and premetallized) and reactive dyes can be used to dye wool fiber. A significant amount of research work has been carried out to understand the thermodynamic and kinetic behavior of nonmetallized acid dyes on wool fiber.^13−15^ The dye fiber substantivity is determined by the interactions present between the protonated amine (NH_3_^+^) groups and the anionic dye molecules. Donnan first proposed the ion exchange theory (electrostatic interactions) of wool dyeing with acid dye, and this was further modified by Gilbert and Rideal to introduce the Langmuir model.^16,17^ The modern theories of dyeing proposed by various researchers reveal the involvement of van der Waals forces, hydrogen bonding, hydrophobic linkages as well as ionic interactions within the dye fiber linkages (Figure 1).^18−20^

Wool fibers from different breeds have differences in both their morphological and chemical properties. The proportion and design of cortical cells in the wool fiber vary with the change in diameter.^21^ The cortical cells consist of the ortho-, para-, and meso-cortex. The ortho- and para-cortex have different compositions providing a unique bilateral structure to the fiber.^5^ The ortho-cortex contains fewer cystine linkages compared to para-cortex and the presence of cystine linkages makes the intermacro fibrils highly cross-linked (less flexible) thus preventing chemicals from reacting with the para-cortex.^22^ This makes the ortho-cortex more chemically reactive.^23^ This phenomenon has been identified by differential staining of the ortho and para-cortex when dyed with basic dyes.^24^ Interestingly the ortho- and para-cortex did not show differential or preferential dyeing when dyed with acid dyes. It was reported that wool fiber in an acid dyeing system did not show difference in dye uptake between Merino and Lincoln wool despite the changes in proportion or geometry of the cortical cells (morphological change).^25^

Acid dyes are anionic dyes adsorbed on the surface of the fiber through the electrostatic attraction between the positively charged amino acids (NH_3_^+^) and dye anion (DSO_3_^–^). If the content of amino acids containing basic groups (lysine, histidine, and arginine) are high in the wool fiber, it will tend to attract more anionic dye molecules during the adsorption stage.^26^ This phenomenon is most evident at an early stage of dye diffusion. Despite variations seen in amino acid content in different breeds of wool, it has been observed that at equilibrium conditions, there is very little difference in the amount of acid dye uptake among wool fibers obtained from different breeds of sheep.^27,28^ However, the reported merino wool fibers were dyed at lower concentration, and to investigate the maximum dye sorption capacity of a new breed of wool fiber, it is required to dye the fiber at higher dye concentrations and temperatures. This could highlight differential dyeing due to presence of different types of scale structure on the surface of the fiber.^29^

Differential dyeing in wool fiber has been reported by modifying the wool fiber characteristics.^30,31^ Researchers have claimed that changes in the amino acid content of wool fiber, obtained from specific breeds, can have an impact on the dyeing properties.^32,33^ El-Nahas in his research focused on how pretreatment of wool fibers with amino acid can increase the dye uptake.^34^ The wool fibers were treated with various concentrations of amino acid solutions before they were dyed with Acid Violet 48. The four types of amino acids used were histidine, phenylalanine, alanine, and valine. These amino acids showed an increase in acid dye exhaustion with an increase in the concentration of treatment and this was observed at very early dyeing times (1–4.5 min).^34^

Modification of the surface cuticle layer of the wool fiber by mechanical and chemical treatment leads to an improvement in the sorption capacity of the fiber. This makes the fiber more accessible to certain chemicals and dyes even at lower treatment temperatures. Differential dyeing for acid dyes on wool fibers obtained from various breeds has seldom been observed.

Wool fibers available in the UK can be investigated by studying the dyeing behavior of the fibers.^1,35^ This paper investigates the difference in dye uptake among wool fibers obtained from different UK sheep breeds, the type of dyeing isotherm they follow, and further analyses of the type of sorption. The thermodynamic parameters of the acid dyes on these wool fibers were calculated to understand the surface behavior of the wool fibers. This research also reported the theoretical acid dye sorption capacity of the wool fibers, which is relevant for the optimization of acid dyeing processes for these wool fibers and enable value-added application where high sorption behavior is demanded. The thermodynamic and kinetic study was investigated to understand the type of sorption of these wool fibers and how the differences in morphological properties can be related to their dyeing behavior.

## 2 Results and Discussion

### 2.1 Difference in Dye Sorption Capacity

All three varieties of wool fiber were dyed, and the [D]_f_ vs [D]_s_ (dye in fiber vs dye in solution) curves were plotted. It was observed that as the concentration of the dye is increased in the dye bath, the amount of dye present in the fiber increases. This means that with a higher amount of dye molecules present in the dye bath, more dye sites in the wool fibers are occupied.

**Figure 2:**
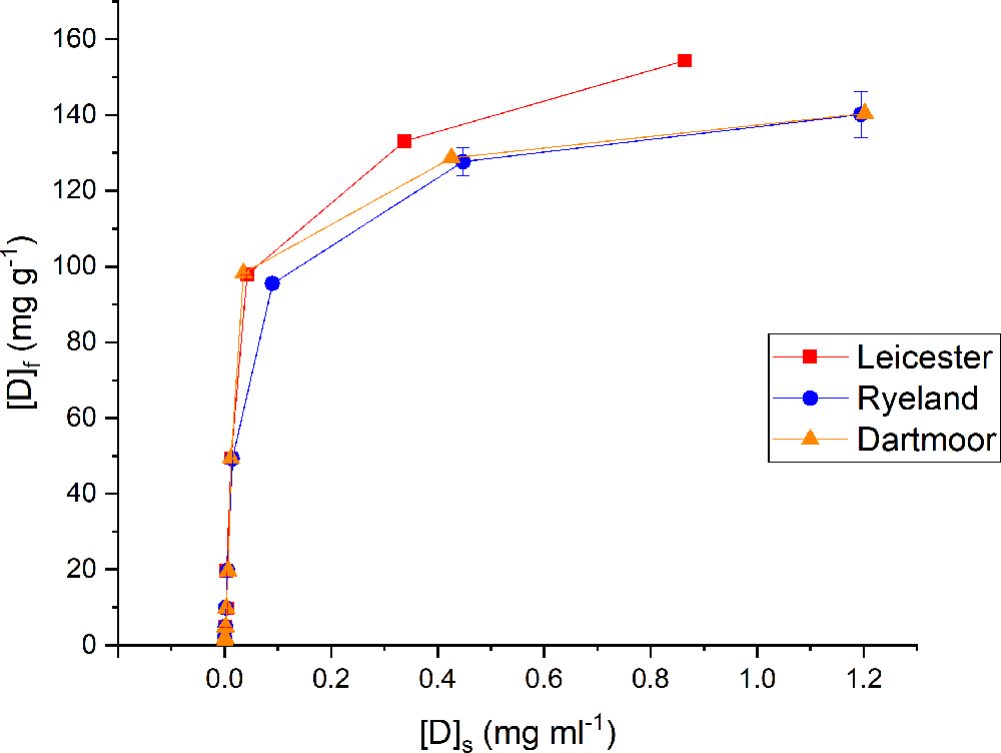
[D]_f_ vs [D]_s_ curves for all the wool fibers (the error bars represent standard error of mean).

When the maximum concentration of the dye was increased to 20% (omf), a significant difference was observed between the dye sorption capacity of Leicester and Dartmoor fibers. The mean experimental dye sorption capacity ([S]_f_) for Leicester and Dartmoor were 154 and 140 mg g^–1^ (Figure 2), and their exhaustion values were 77 and 70%, respectively. Finer fiber is associated with larger surface area compared to a coarser fiber, which can lead to a higher dye uptake, but this phenomenon is observed in synthetic fibers where the linear density of the fiber is almost constant.^36^ Wool is a very complex fiber, which does not possess a uniform diameter and has different scale patterns on the fiber surface, which could influence the dyeing sorption properties.^29^ The measurement methodology and results of mean fiber diameters of Leicester and Dartmoor have been reported by the authors in an earlier publication.^29^ Leicester is a finer fiber (mean fiber diameter 22 μm), Ryeland (31 μm) being a medium, and Dartmoor is a coarser fiber; the difference in surface area of the fibers could be one of the reasons for the difference in experimental dye uptake. There may be other factors involved like differences in surface properties, morphological structure of the fibers, or amino acid composition of the fibers.^15^ The involvement of scale and surface morphology of the wool fiber in dyeing kinetics has been investigated in detail in an earlier publication by the authors.^29^ At the same time, at a higher concentration of dye, the sorption behavior may change depending on amino acid chemistry, i.e., the composition of basic amino acids (arginine, lysine, and histidine) within the wool fiber.^37^

### 2.2 Dyeing Thermodynamics

**Figure 3:**
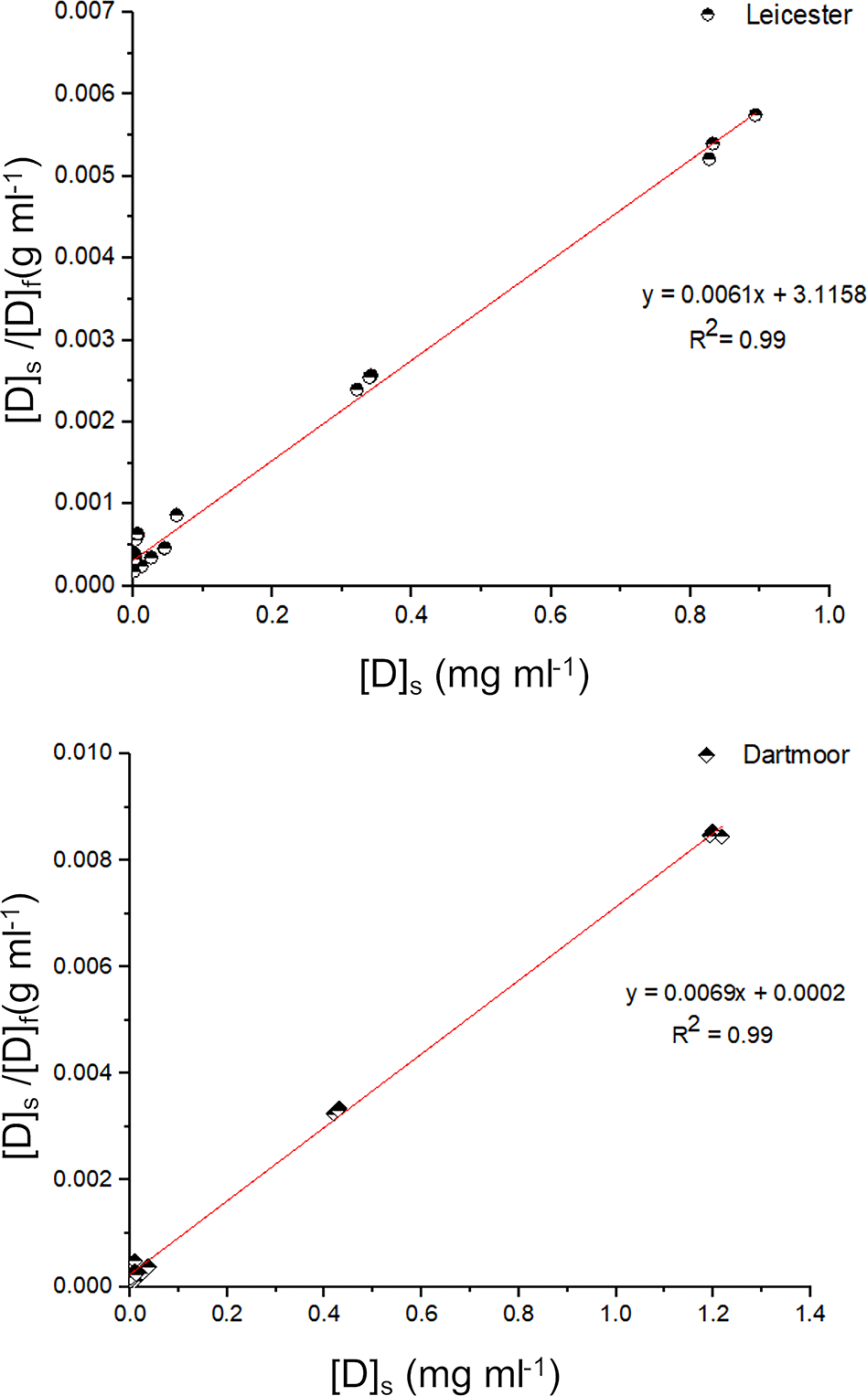
Plot of [D]_s_/[D]_f_ vs [D]_s_ Langmuir adsorption isotherm for Leicester and Dartmoor wool fibers.

**Figure 4:**
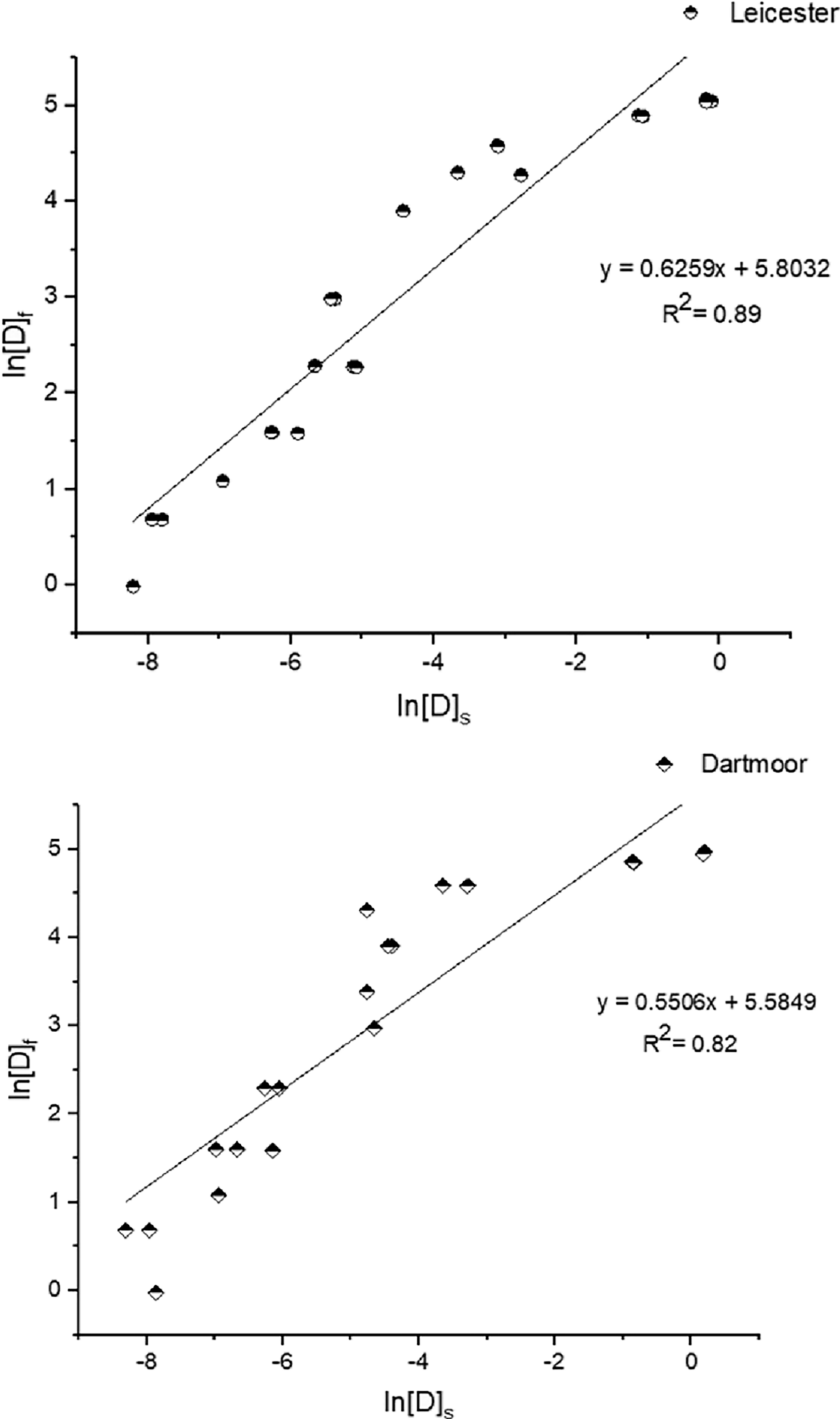
Plot of ln[D]_f_ vs ln[D]_s_ Freundlich adsorption isotherm curve for Leicester and Dartmoor wool fibers.

The experimental data of dye sorption of Leicester and Dartmoor were fitted against Langmuir and Freundlich isotherms. Ryeland showed an observable difference in dye uptake above 10% concentration, and due to this, it has been excluded in the thermodynamics discussion. The straight-line plots showing the correlation (*R*^2^) between Langmuir and Freundlich isotherm for Leicester and Dartmoor wool are shown in Figures 3 and 4. Table 1 shows the comparative data of both the isotherms and their respective constants for the fibers.

**Table 1:** Comparison between the Langmuir and Freundlich thermodynamic constants and parameters

	Langmuir		
wool fiber breed	[S]_f_(mg g^−1^)	*K*_L_ (mL mg^−1^)	*R*^2^
Leicester	164	20	0.99
Dartmoor	144	35	0.99

	Freundlich		
	*K*_F_ (mL mg^−1^)	*N*_F_ = 1/*n*	*R*^2^
Leicester	342	1.58	0.89
Dartmoor	285	1.77	0.82

The *R*^2^ (0.99) values of the linearized plots associated with Langmuir isotherm are higher than those associated with linear plots of Freundlich isotherm, for Leicester and Dartmoor wool fibers. Thus, both the wool fibers follow Langmuir isotherm model for the adsorption of Acid Red 1. Theoretically, Langmuir isotherm is associated with monolayer adsorption. The adsorption process take place due to the electrostatic interactions between the anionic dyes and the protonated amine groups of wool fiber in an aqueous medium.

The respective *R*^2^ values of the Freundlich isotherm model for Leicester and Dartmoor fibers are low (0.89, 0.82) compared to the Langmuir suggesting a relatively poor fit of the experimental data against the Freundlich isotherm model.

The *K*_L_ and the [S]_f_ value for Langmuir was obtained from the slope and intercept of the linear plot shown in Figure 3. The theoretical dye sorption capacity values ([S]_f_) for Leicester and Dartmoor are 164 and 144 mg g^–1^, respectively. The respective *K*_L_ values for Leicester and Dartmoor are 20 and 35 mL mg^–1^. This signifies that Dartmoor wool fibers have a stronger interaction with Acid Red 1 as compared to Leicester.^38^

### 2.3 Thermodynamic Parameters

The standard affinity (Δμ°) values calculated for Leicester and Dartmoor using eq 7 are −19.30 and −17.94 kJ mol^–1^ (Table 2). The standard affinity is associated with the tendency of a fiber to adsorb the dyes. This result shows that the standard affinity for Leicester is more than Dartmoor, which suggests a higher ability of dye molecules to migrate from the solution to the Leicester fiber.^39,40^

**Table 2:** Comparison of Standard Affinity and Adsorption Energy of Leicester and Dartmoor Fibers

wool breeds	Δμ° (kJ mol^–1^)	Δ*G*° (kJ mol^–1^)
Leicester	–19.30	–4.03
Dartmoor	–17.94	–2.59

The adsorption energy (Δ*G*°) values for Leicester and Dartmoor are −4.03 and −2.59 kJ mol^–1^ (Table 2); the negative value implies that the thermodynamic system is spontaneous and moving toward equilibrium. This also suggests that Dartmoor is closer to the theoretical dye sorption capacity (Δ*G*° = 0) as compared to Leicester.

The current standard affinity values show Leicester (22 μm) have a higher standard affinity (Δμ°) than Dartmoor (72 μm). This is in agreement to the established fiber dyeing theory that finer fibers have higher affinity compared to coarser fibers.^41,42^

### 2.4 Dyeing Kinetics

**Figure 5:**
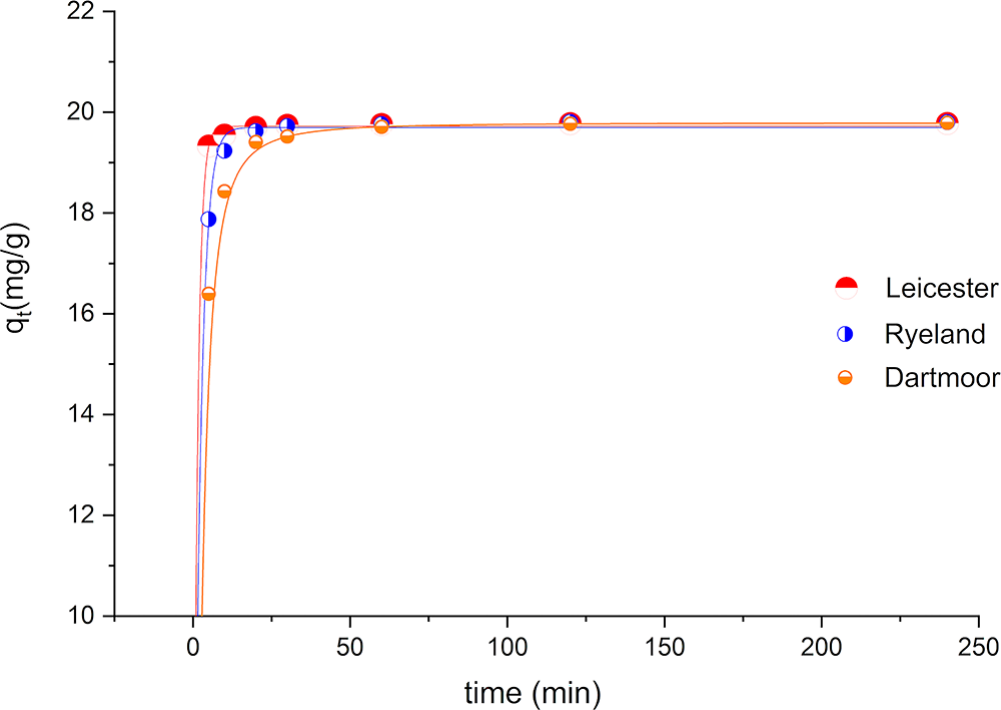
*q_t_* vs time graph for Leicester, Ryeland, and Dartmoor wool fiber.

Amount of dye adsorbed by the fiber at time *t* (*q_t_*) is expressed as a function of time (min) in Figure 5 for Leicester, Ryeland, and Dartmoor. Ryeland is included in this study, as at a lower dye concentration (2% omf), it did not show any observable variation in dye uptake.

The adsorption of Acid Red 1 on all the fibers shows a gradual and nonlinear increase with time and finally reaches a plateau. At the initial stages of the dyeing process, the rate of sorption is different for different fibers, but as they reach equilibrium, the adsorption value becomes constant for all the fibers (∼19.8 mg g^−1^) (99% exhaustion @ 2% omf).

**Figure 6:**
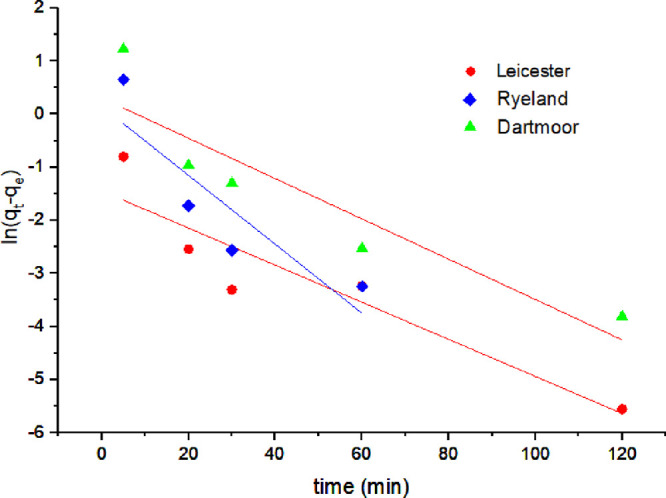
Pseudo-first-order kinetics {ln(q_t_-q_e_) vs time} linear plots for Leicester, Ryeland, and Dartmoor wool fibers.

**Figure 7:**
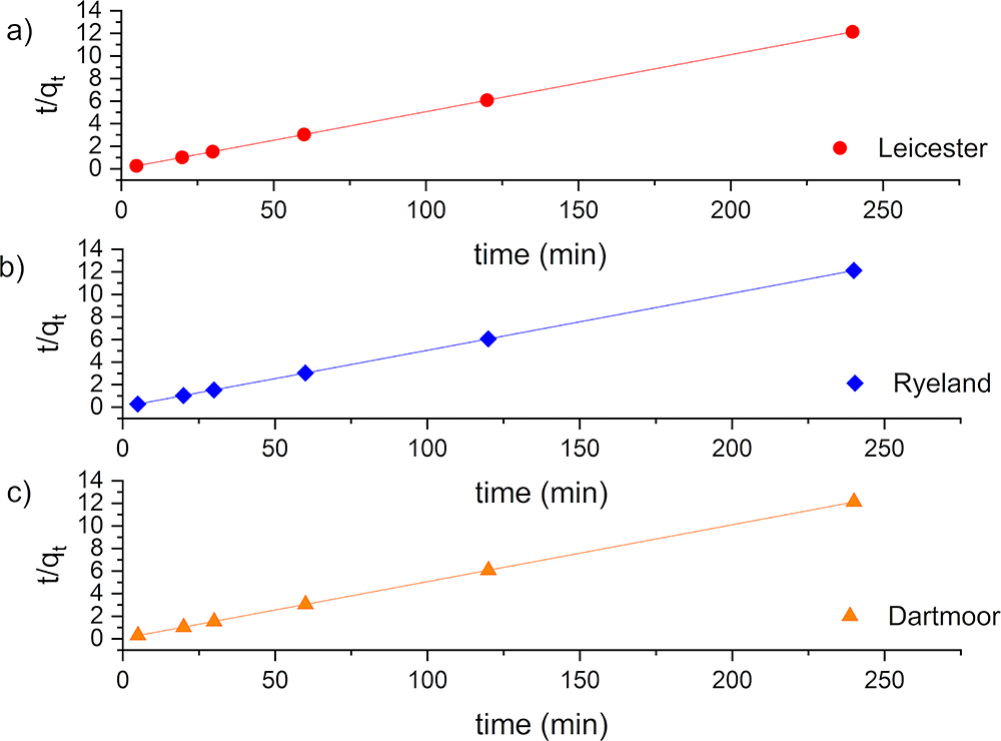
Pseudo-second-order kinetic {*t*/*q_t_* vs time} linear plots for (a) Leicester, (b) Ryeland, and (c) Dartmoor.

The dyeing kinetics data were compared against pseudo-first and second-order kinetic models to understand the adsorption process of dyes, mass transfer, and reaction speed between the dye and fibers.^43^ The rate constants (*k*_1_, *k*_2_), *q*_e_, and correlation coefficients from the linearized plots are shown in Table 3. The linearized plots of the respective models are shown in Figures 6 and 7. The correlation coefficient (*R*^2^) values for the pseudo-first-order kinetic reaction are low compared to the pseudo-second-order kinetic reaction for all the fibers (Table 3).

**Table 3:** Pseudo-First Order and Second-Order Rate Constants, *q*_e_, and *R*^2^ Values for Leicester, Ryeland, and Dartmoor Wool Fibers

	pseudo-first-order			pseudo-second-order		
breed	*k*_1_ (min^–1^)	*q*_e_ (mg g^–1^)	*R*^2^	*k*_2_ (mg g^–1^ min^–1^)	*q*_e_ (mg g^–1^)	*R*^2^
Leicester	0.03	0.23	0.81	0.50	19.80	1.00
Ryeland	0.06	1.15	0.67	0.18	19.83	1.00
Dartmoor	0.04	1.35	0.79	0.08	19.84	1.00

High *R*^2^ (1.00) values were obtained from the linearized plot of pseudo-second-order kinetic model for all the fibers. Also, the calculated values of *q*_e_ for all the fibers are in good agreement with the theoretical values. This suggests Leicester, Ryeland, and Dartmoor follow pseudo-second-order kinetic model confirming monolayer sorption as the rate-determining step.^44^

Ryeland also followed pseudo-second-order with Acid Red 1 signifying that at lower concentrations the mobility of the dye molecules will be high and the electrostatic bond formation with the NH^3+^ sites of the wool fiber will be rapid.^15^ The pseudo-second order rate constants can be ranked *k*_2_ (Leicester) > *k*_2_ (Ryeland) > *k*_2_ (Dartmoor) (Table 3). The rate constant value signifies how fast or slow the reaction will proceed.^45^ This means that Acid Red 1 dye will migrate toward Leicester at a faster rate (from solution to the fiber core) as compared to Ryeland and Dartmoor.

#### 2.4.1 Time of Half Dyeing

**Figure 8:**
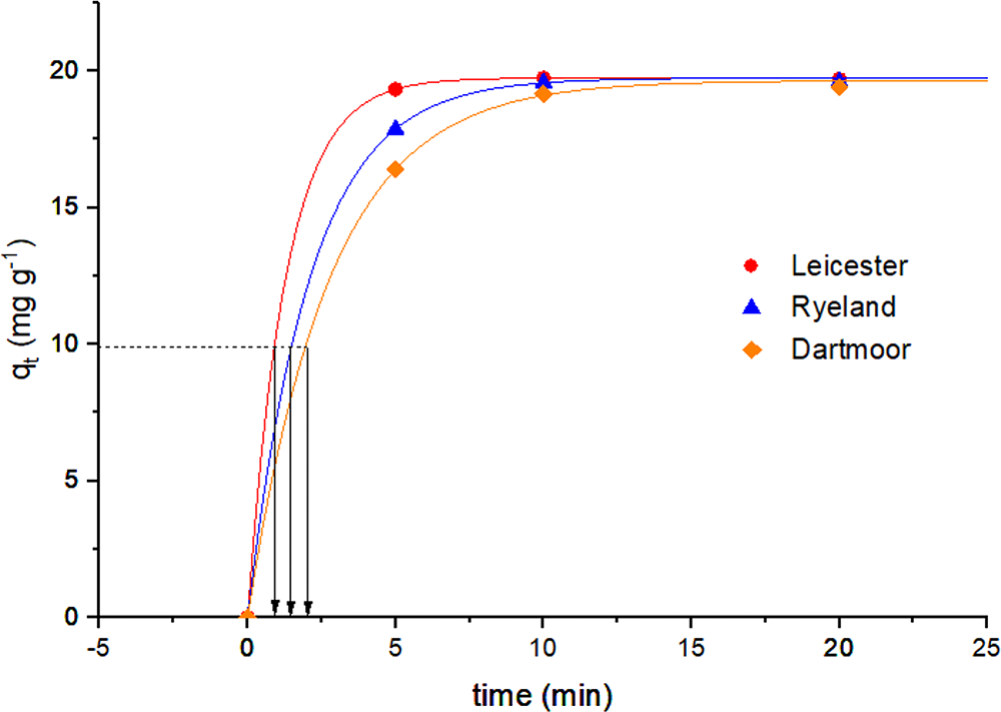
Time of half dyeing calculation for Leicester, Ryeland, and Dartmoor wool fibers.

The time of half dyeing (*t*_1/2_) was calculated for all the fibers as shown in Figure 8 and reported in Table 4. Leicester has the fastest rate of dye sorption, as the *t*_1/2_ is reported as 0.9 min. This means that 50% of Acid Red 1 dye present in the solution have migrated to the fiber after 0.9 min. Similarly, for Ryeland and Dartmoor, the *t*_1/2_ values were reported as 1.5 and 1.9 min, respectively. Thus, the ranking of the rate of Acid Red 1 dye adsorption can be expressed as Leicester > Ryeland > Dartmoor. These results were in concurrence with the pseudo-second-order rate constant.

**Table 4:** Time of Half Dyeing Values of Leicester, Ryeland, and Dartmoor Wool Fibers^29^

breeds	mean fiber diameter (μm)	*t*_1/2_ (min)	exhaustion (%)
Leicester	22	0.9	99
Ryeland	31	1.5	99
Dartmoor	72	1.9	99

The time of half dyeing (*t*_1/2_) and the mean fiber diameter of the wool fibers suggests that with an increase in mean fiber diameter there is an increase in the *t*_1/2_ value (Table 4). Finer fibers have a higher surface area, and they reach dye equilibrium quickly as compared to coarser fibers.^42^ This might be the reason behind a lower *t*_1/2_ value for Leicester wool fibers. Coarser fibers have a lower surface area and take more time to dye, which is the reason for a higher *t*_1/2_ value for Ryeland and Dartmoor. This theory of fiber diameter is more accurate for fibers that are circular in cross section, whereas wool fiber itself varies in the shape as well as the diameter of the fiber.^46^ The presence of scales on the surface of the wool fiber makes it irregular in shape (noncircular cross section) and variable in terms of diameter within a single fiber. No observable difference in dye uptake were observed among Leicester, Ryeland, and Dartmoor for the kinetics study (at 2% omf). All the fibers had a *q*_e_ value of ∼19.80 mg g^−1^. This similar trend was also observed in thermodynamics study of all three fiber. No observable difference in dye uptake was observed at 10% concentration (omf) (Section 2.1 and Table S1 (Supporting Information). Diameter could not be the only factor responsible for the difference in dye uptake observed between Leicester and Dartmoor (at 20% omf), as its effect was not replicated at lower concentrations (2 and 10% omf) where the exhaustions are 99 and 98%.

### 2.5 Discussion

The authors have previously established that changes in scale pattern can affect the diffusion rate of an acid dye.^29^ In spite of the difference in scale pattern, diameter, amino acid composition, and rate of dyeing (time of half dyeing), no observable difference was seen between the dye sorption capacity values (at 2 and 10% omf) among the wool fibers.

**Figure 9:**
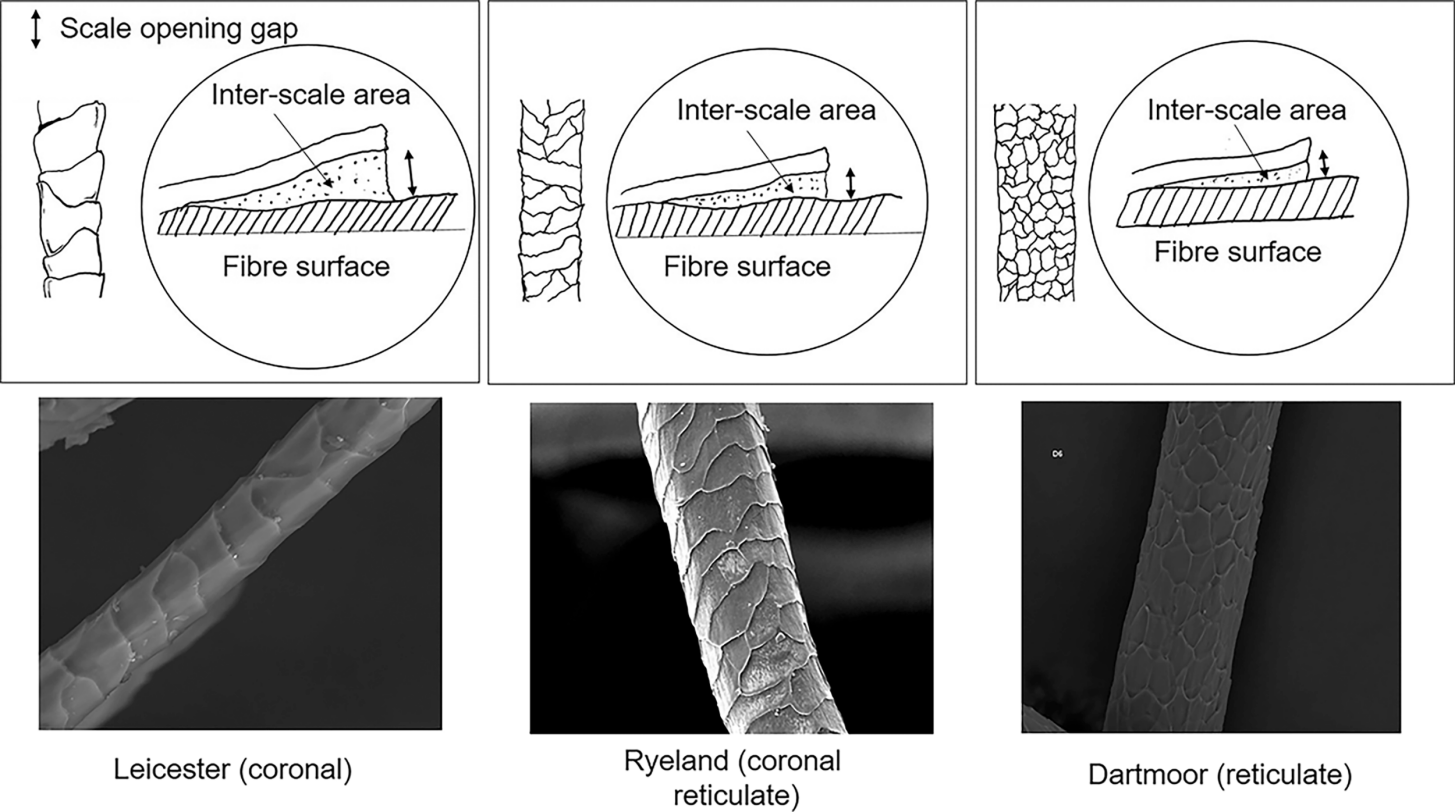
Schematic diagram representing change in scale opening gap and interscale area for Leicester, Ryeland, and Dartmoor wool fibers. Adapted with permission from ref (29). CC BY 4.0 https://creativecommons.org/licenses/by/4.0/.

It was also reported that as the diameter of the fiber increases from Leicester to Ryeland to Dartmoor, the scale pattern shifts from coronal to coronal reticulate to reticulate.^29^ It can be proposed that as the scale pattern changes from coronal to reticulate, the area of the diffusion pathway decreases. Finer fibers are mostly associated with a coronal scale pattern where the scales overlap on each other, creating more area for the dye to diffuse (Leicester). The dye can diffuse through the scale opening gaps created between the surface of the fiber and the scale edge. The crucial aspect is the interscale area, which is predicted to be much higher in case of coronal type patterns as shown in schematic diagram in Figure 9. The increase in diameter of the fiber changes this pattern of scale from coronal to coronal reticulate (Ryeland), and a greater number of scales are visible on the surface. Simultaneously the scale opening gap between the fiber surface decreases (Figure 9). This may result in a decrease in interscale area, thus allowing a less accessible path for the dyes. Similarly, as the pattern shifts to coarser fibers (Dartmoor), the reticulate scales appear to be highly packed; thus, it can be proposed that there is a further decrease in the scale opening gap and interscale area for the dyes to diffuse through (Figure 9).

An interpretation of the dyeing thermodynamics and kinetics result is that the molecular size of the Acid Red 1 dye was small enough to penetrate through the scale opening gap of all the different types of fibers with different scale patterns to provide similar exhaustion values (∼98%) at 2 and 10% omf. The difference in dye uptake observed at 20% omf can be a combined effect of the change in the scale opening gap and the molecular size of the dye. The scale opening gap in Leicester can be assumed to be greater than that in Dartmoor. Thus, Leicester has a more accessible path for the diffusion of dye molecules, which can result in a higher dye uptake than Dartmoor. At the same time, the standard affinity of Acid Red 1 and the *k*_2_ values of Leicester is higher than Dartmoor. This suggests Acid Red 1 migrates quickly to the Leicester wool fiber and gets adsorbed on the dye sites of the wool fiber. At a higher concentration, the amount of dye molecules in the dye bath increases and as they reach the surface of the fiber, there could be a possibility of a higher number of interaction between the dye molecules.^13^ This can result in an increase in dye dimension and thus agglomeration of dye molecules on the surface of the fiber. Leicester was predicted to have a higher scale opening gap due to its circular scale structure, and Dartmoor was predicted to have a small scale opening gap due to its packed scale structure. Thus, the dye molecules at higher concentration (above 10% omf) showed a lesser dye uptake value for Dartmoor (140 mg g^–1^) as compared to Leicester (154 mg g^–1^). The discussion suggests that scale opening gap is an important factor which affects the dye uptake of wool fibers.

## 3 Conclusions

This paper confirms the existence of a difference in the dye uptake values between wool fibers obtained from different breeds. The mean experimental dye sorption capacity for Leicester and Dartmoor from the [D]_f_ vs [D]_s_ curves are reported to be 154 and 140 mg g^–1^ at 20% omf, respectively. The difference in the exhaustion of CI Acid Red 1 dye between Leicester and Dartmoor is 7%. Ryeland wool showed the presence of color variation within the fleece which resulted in variation of the dye uptake values at 20% omf.

The dyeing isotherm data was fitted against Langmuir and Freundlich isotherm models. It was concluded from the *R*^2^ (0.99) values of the Langmuir plots that both Leicester and Dartmoor follow a Langmuir isotherm. Theoretical dye sorption capacities for Leicester and Dartmoor were calculated from the model which was 164 and 144 mg g^–1^, respectively. Thermodynamic factors like separation factor (*R*_L_), adsorption energy (Δ*G*_o_), and standard affinity of dyeing (Δμ_o_) were also calculated to understand the sorption behavior. The magnitude of the standard affinity (Δμ_o_) is higher for Leicester (−19.30 kJ mol^–1^) compared to Dartmoor (−17.94 kJ mol^–1^). The adsorption energy values suggested that both the isotherms are thermodynamically favorable.

The adsorption kinetic models for Leicester, Ryeland, and Dartmoor were investigated, and they followed pseudo-second-order reaction kinetics. The change in rate of dyeing for the wool fibers were reflected by the difference in time of half dyeing. The discussion on the scale morphology suggested that despite the differences observed in time of half dyeing, no change in dye sorption capacities were observed at 2 and 10% omf. The thermodynamics study suggested that the scale opening gap could be an influential factor, which determines the dye sorption capacity of the wool fibers. This research work unfolds further opportunities to investigate the effect of scale opening gap on dyeing kinetics of acid dyes on wool fibers.

## 4 Materials and Methodology

### 4.1 Materials

Wool fibers from three different UK sheep breeds, Bluefaced Leicester, Ryeland, and Greyface Dartmoor were obtained from the Fleet Green Farm, Lancaster. For the pretreatment of wool fibers, sodium carbonate (Na_2_CO_3_), sulfuric acid (H_2_SO_4_), and ULTRAVON JUN (a nonionic detergent) were used as received. CI Acid Red 1 (60% dye content), sulfuric acid, and sodium sulfate (Na_2_SO_4_) were used for dyeing of wool fibers. All chemicals were supplied by Sigma-Aldrich, except ULTRAVON JUN, which was manufactured by Huntsman Textile Effects (Germany) GmbH and supplied by Town End (Leeds). Deionized water was used for preparing all the dye solutions.

### 4.2 Methodology

#### 4.2.1 Scouring of Wool

Wool scouring was performed by a five-bath process using the recipe given in Table 5.^47^ Wool fibers were taken from the fleece randomly to avoid preferential sampling. Twenty g samples of wool fiber were scoured at a liquor ratio of 1:50 with constant manual agitation. When the fibers were transferred from one bath to another, they were squeezed first to reduce the transfer of liquor from one bath to the next. All the fiber samples were then rinsed in an excess of cold water and squeezed twice before drying at 90 °C for 1 h in a convection oven (BINDER). The fiber samples were conditioned at 20 ± 2 °C and 65 ± 3% RH for 24 h before further treatment.

**Table 5:** Recipe for Wool Scouring

recipe	1st bath	2nd bath	3rd bath	4th bath	5th bath
Na_2_CO_3_	1.33 g L^–1^	0.65 g L^–1^	0.65 g L^–1^		
ULTRAVON JUN	7.5 g L^–1^	5.0 g L^–1^	2.5 g L^–1^		
temperature	60 °C	55 °C	50 °C	45 °C	45 °C
time	3 min	3 min	3 min	3 min	3 min

#### 4.2.2 Carbonizing of Wool and Vegetable Matter

The scoured wool samples were carbonized based on a modified recipe reported by Park.^48^ The wool fibers were immersed in the solution of liquor ratio of 1:20, which consists of sulfuric acid (70.0 g L^–1^) and ULTRAVON JUN (2.0 g L^–1^), for 2 h at 20 ± 2 °C. The pH of the solution was 1.2. The fibers were then removed, rinsed in cold water, dried at 80 °C for 1 h, and finally baked at 100 °C for 10 min. After baking, the fibers were placed on a table and manually crushed with a metal roller so that the impurities and the vegetable matters were easily removed. The wool fibers were rinsed once in cold water and then washed in a solution (1:20 liquor ratio) of sodium carbonate (53.0 g L^–1^) at room temperature. They were rinsed in cold water, squeezed, and then dried overnight at room temperature.

#### 4.2.3 Dyeing of Wool

For the thermodynamics study, 1.0 g wool fibers from Bluefaced Leicester, Ryeland, and Greyfaced Dartmoor were dyed using CI Acid Red 1 of varying concentrations of 0.1, 0.2, 0.5, 1.0, 2.0, 5.0, 10.0, 15.0, and 20.0% on mass of fiber (omf). The dyeing was performed in a Roaches Pyrotec IR dyeing machine with a material:liquor ratio of 1:50. 1.0 ± 0.2% omf H_2_SO_4_ was used to maintain the pH, and 5.0% omf Glauber’s salt was used as a leveling agent. The pH of the dyeing solution was maintained at 2.7 ± 0.2, dyeing temperature 90 °C, and dyeing time 90 min.

For kinetics study, 1.0 g of wool fibers was dyed using 2.0% (omf) Acid Red 1 dye, 5.0% (omf) Glauber’s salt, and 1.0 ± 0.2% (omf) sulfuric acid. The dyeing time was varied from 5 to 10, 20, 30, 60, 120, and 240 min. The dyeing temperature was maintained at 90 °C. The dyeing profile for thermodynamic and kinetic study is provided in the Supporting Information (Figure S1). The remaining dye liquors from the dye tubes were collected in glass vials and cooled to room temperature before measuring the absorbency of the samples. The experimental data of all the thermodynamic and kinetic studies are provided in Tables S1 and S2 (Supporting Information).

#### 4.2.4 Dyeing Adsorption Isotherms

Dye solutions left after the completion of dyeing were used to measure the absorbance values using a UV visible spectrophotometer (Jasco V-730). The absorbance of the residual dye solution was calculated to be 532 nm (λ_max_) (Figure S2).

The amount of dye adsorbed at any time [D]_f_ (mg g^–1^) was determined by the equation below.
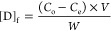
1where *C*_o_ is the initial dye concentration (mg mL^–1^) in the solution, *C*_e_ is the left-over dye concentration at equilibrium (mg mL^–1^), *V* is the initial volume of the solution (mL), and *W* is the weight of wool fiber (g).

##### Langmuir Isotherm

The Langmuir isotherm model assumes that adsorption happens on a homogeneous adsorbent surface when each site occupies an identical number of adsorbents and that they are equivalent in terms of energy. The model also assumes that the adsorbent does not interact with any adjacent sites. Theoretically, there is a finite capacity of adsorbate molecules on the adsorbent surface (monolayer adsorption).^49^ After the adsorbent surface is saturated, no further adsorption takes place, thus making it an irreversible process. This isotherm is demonstrated by the following equation:
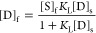
2where [D]_f_ and [D]_s_ are respective dye concentrations in fiber and solution and *K*_L_ (mL mg^–1^) and [S]_f_ (mg g^–1^) are the Langmuir constants. The calculated concentration of dye in the fiber at equilibrium (mg g^–1^) is the saturation dye uptake ([S]_f_).

The original equation of Langmuir model (eq 2) was rearranged as follows:^50^
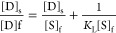
3

During the rearrangement, it was assumed that [D]_f_ = [S]_f_ at equilibrium.

A straight line of best fit was plotted against [D]_s_/[D]_f_ vs [D]_s_ to calculate *K*_L_ and [S]_f_ from the slope and *y*-intercept, respectively.

##### Freundlich Isotherm

The Freundlich isotherm model is based on the assumption that adsorption takes place on surfaces that are heterogeneous in nature and on sites that have very different adsorption energies. The adsorption process is dependent on the concentration of the adsorbate in the solution.^49^ The amount of adsorbate adsorbed on the surface increases with an increase in concentration. The Freundlich isotherm is characterized by multilayered adsorption. The isotherm is demonstrated by the following equation:
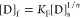
4where *K*_f_ is the Freundlich constant, and 1/*n* is the heterogeneity factor.

The original equation of Freundlich’s model (eq 4) can be rearranged and expressed as
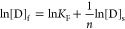
5

A straight line of best fit can be plotted against ln[D]_f_ and ln[D]_s_ to give the values of 1/*n* and *K*_F_ from its slope and *y*-intercept representing the adsorption intensity and capacity, respectively. If *n* > 1, then the adsorption isotherm becomes favorable.

#### 4.2.5 Thermodynamic Parameters

Gibb’s free energy (Δ*G*°), also known as adsorption energy for the dye fiber system, was calculated from eq 6.^51^
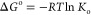
6where *K*_o_ is the solid phase concentration at equilibrium (mg mL^–1^)/liquid phase dye concentration (mg mL^–1^) at equilibrium, *R* is the universal gas constant (8.314 J mol^–1^ K^–1^), and *T* is the temperature (Kelvin).

Standard affinity of dyeing is defined as the capability of the dye to move from the solution phase to the fiber.^52^ This can be calculated from eq 7.
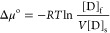
7where Δμ° is the standard affinity of the dye for the fiber in kJ mol^–1^, *R* is the universal gas constant (8.314 J mol^–1^ K^–1^), *T* is the temperature (Kelvin), and *V* is the effective volume of water in a dry fiber; the theoretical value of this is taken as 0.31 mL g^–1^ for standard affinity calculations for wool fibers.^52,53^

#### 4.2.6 Dyeing Kinetic Models

The amount of Acid Red 1 dye adsorbed on the fiber was calculated using eq 8
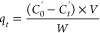
8where *q_t_* is the amount of dye adsorbed in mg g^–1^ at any time (*t*) (min), *C*_o_′ (mg mL^–1^) is the initial dye concentration, *C*_t_′ is the dye concentration (mg mL^–1^) at time *t*, *V* is the volume of the dye solution, and *W* is the weight of the wool fiber samples.

The amount of Acid Red 1 adsorbed on the fiber at equilibrium was calculated using eq 9
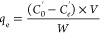
9where *q*_e_ is the amount of dye adsorbed in mg g^–1^ at equilibrium, C_e_′ is the dye concentration (mg mL^–1^) at equilibrium.

The pseudo-first-order kinetic model equation can be represented by eq 10
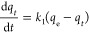
10

A linearized form of eq 10 can be expressed as
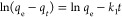
11

A straight line plot between ln(*q*_e_ – *q_t_*) and *t* enables the values of *k*_1_ (rate constant) and *q*_e_ to be calculated from the slope and *y*-intercept, respectively.^54^

The pseudo-second-order kinetic model can be represented by eq 12
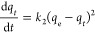
12

A linearized form of eq 12 can be expressed as
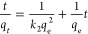
13

A straight line plot between *t*/*q_t_* and *t* gives the values of *k*_2_ (rate constant) and *q*_e_ (calculated from the model) from the slope and *y*-intercept.^54^

## Supporting Information Available

Figures of dyeing profile and calibration curve of Acid Red 1 dye and tables of dyeing thermodynamics and kinetics experimental data of the wool fibers (PDF)

## Notes

The Supporting Information contain the relevant experimental data whereas any other requirement of data are available upon reasonable request to the corresponding author.
