# Supplementary material for: Investigation of the Thermodynamic and Kinetic Behavior of Acid Dyes in Relation to Wool Fiber Morphology
Source: ACS Omega. 2024 Jun 4;9(24):25922–31. doi: 10.1021/acsomega.4c00560 (PMC11191121; doi:10.1021/acsomega.4c00560)
Supplement: Supplementary file 1 — ao4c00560_si_001.pdf [file ao4c00560_si_001.pdf]

Supplementary material

Investigation of the thermodynamic and kinetic behaviour of acid dyes in relation to wool fibre morphology

Subhadeep Paul[a,*], Andrew Hewitt[a], Sohel Rana[b] and Parikshit Goswami[a]

[a] Technical Textiles Research Centre, School of Arts and Humanities, University of Huddersfield, Queensgate, Huddersfield HD1 3DH, UK

[b] Department of Textile & Fibre Engineering, Indian Institute of Technology Delhi, Hauz Khas, New Delhi – 110016, India

A.Hewitt@hud.ac.uk , sohelrana@iitd.ac.in , P.Goswami@hud.ac.uk

*Corresponding author- Subhadeep.Paul@hud.ac.uk

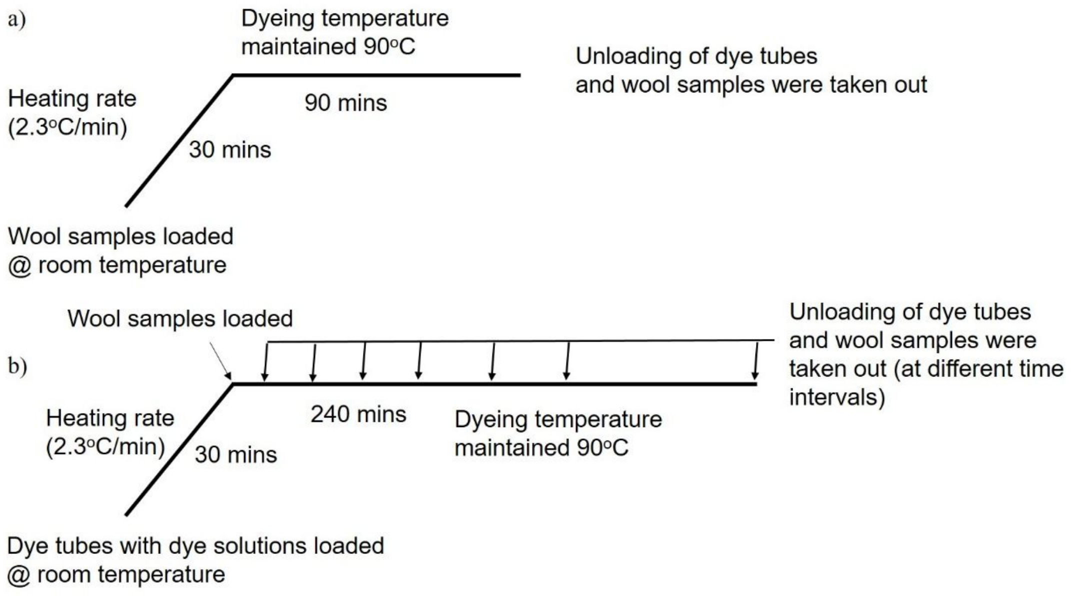

Figure S1: Dyeing profile for a) thermodynamics and b) kinetics studies of wool fibre with Acid Red 1 dye in Roaches IR Dyeing machine.

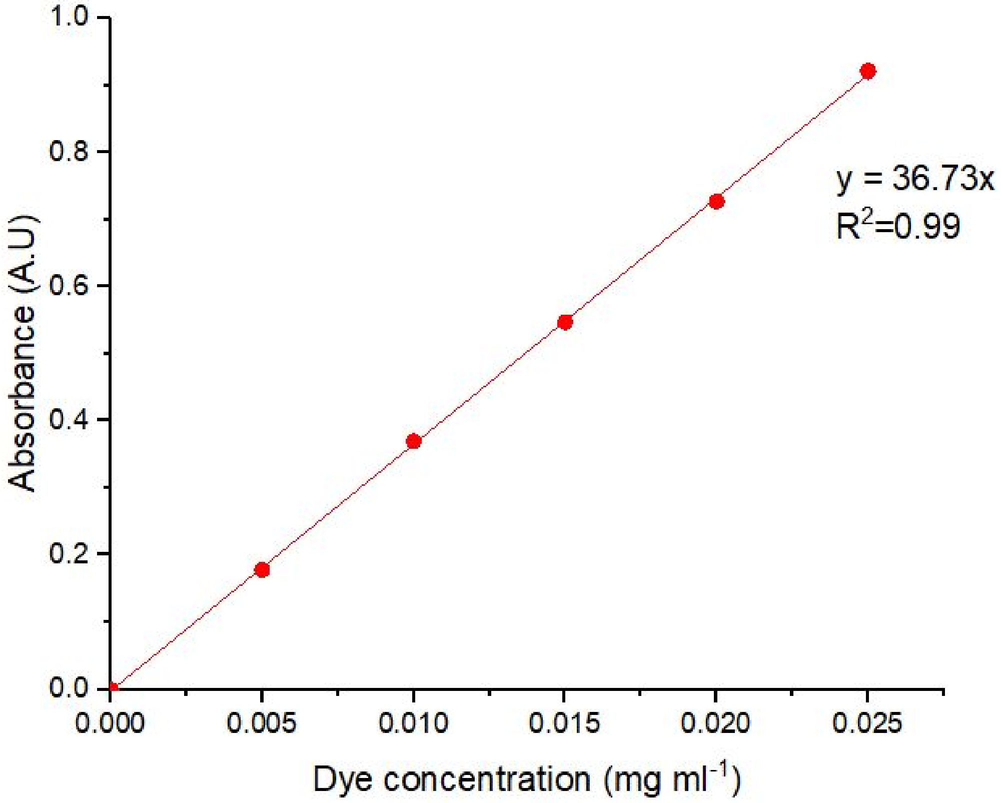

Figure S2: Calibration curve for Acid Red 1 dye

Table S1: Repeat dyeing thermodynamics data of Acid Red 1, $[D]_s$ and $[D]_f$ of all wool fibres

		Set 1		Set 2		Set 3	
**Breeds**	**Conc. of dye (%)**	**$[D]_s$ (mg ml^{-1})**	**$[D]_f$ (mg g^{-1})**	**$[D]_s$ (mg ml^{-1})**	**$[D]_f$ (mg g^{-1})**	**$[D]_s$ (mg ml^{-1})**	**$[D]_f$ (mg g^{-1})**
**Leicester**	**0.1**	0.0003	0.99	0.0007	0.97	0.0003	0.98
	**0.2**	0.0004	1.98	0.0009	1.95	0.0004	1.98
	**0.5**	0.0019	4.91	0.0027	4.86	0.0019	4.90
	**1**	0.0059	9.70	0.0062	9.69	0.0035	9.83

	**2**	0.0043	19.78	0.0041	19.79	0.0046	19.77
	**5**	0.0119	49.40	0.0137	49.32	0.0119	49.40
	**10**	0.0445	97.78	0.0371	98.14	0.0445	97.78
	**15**	0.3391	133.05	0.3326	133.42	0.3415	132.86
	**20**	0.8321	154.12	0.9336	153.31	0.8169	155.75
		**Set 1**		**Set 2**		**Set 3**	
**Ryeland**	**0.1**	0.0005	0.98	0.0003	0.99	0.0003	0.99
	**0.2**	0.0009	1.96	0.0004	1.98	0.0004	1.98
	**0.5**	0.0014	4.93	0.0014	4.93	0.0014	4.93
	**1**	0.0028	9.86	0.0025	9.87	0.0025	9.87
	**2**	0.0058	19.71	0.0054	19.73	0.0053	19.73
	**5**	0.0183	49.08	0.0130	49.35	0.0128	49.36
	**10**	0.0806	95.97	0.0964	95.18	0.0931	95.34
	**15**	0.3488	130.05	0.3988	132.62	0.5965	120.38
	**20**	1.1342	143.47	1.0325	148.52	1.4252	128.32
		**Set 1**		**Set 2**		**Set 3**	
**Dartmoor**	**0.1**	0.0006	0.97	0.0004	0.98	0.0003	0.98
	**0.2**	0.0006	1.97	0.0009	1.96	0.0003	1.98
	**0.5**	0.0021	4.89	0.0013	4.94	0.0021	4.89
	**1**	0.0037	9.81	0.0023	9.88	0.0034	9.86
	**2**	0.0071	19.65	0.0044	19.78	0.0095	19.52
	**5**	0.0134	49.29	0.0123	49.38	0.0115	49.42
	**10**	0.0258	98.71	0.0442	97.79	0.0373	98.13
	**15**	0.4312	128.43	0.4193	129.03	0.4272	128.63
	**20**	1.1974	140.01	1.1923	140.89	1.1983	140.41

Table S2: Repeat Acid Red 1 dyeing kinetics data for $[D]_f$ data set

		Set 1	Set 2
**Breeds**	**Time of dyeing (min)**	**$[D]_f$ (mg/ml)**	**$[D]_f$ (mg/g)**
**Leicester**	**5**	19.33	19.31
	**10**	19.51	19.52
	**20**	19.70	19.71
	**30**	19.74	19.73
	**60**	19.76	19.76
	**120**	19.78	19.78
	**240**	19.78	19.78
		**Set 1**	**Set 2**
**Ryeland**	**5**	17.87	17.84
	**10**	18.93	18.91
	**20**	19.62	19.63
	**30**	19.72	19.70
	**60**	19.76	19.75
	**120**	19.80	19.80
	**240**	19.80	19.80
		**Set 1**	**Set 2**
**Dartmoor**	**5**	16.40	16.32

	**10**	18.42	18.40
	**20**	19.41	19.45
	**30**	19.52	19.56
	**60**	19.71	19.72
	**120**	19.77	19.78
	**240**	19.79	19.80
